# Supplementary material for: Ton motor conformational switch and peptidoglycan role in bacterial nutrient uptake
Source: Nat Commun. 2024 Jan 6;15:331. doi: 10.1038/s41467-023-44606-z (PMC10771500; doi:10.1038/s41467-023-44606-z)
Supplement: Supplementary file 1 — Supplementary Information [file 41467_2023_44606_MOESM1_ESM.pdf]

Supplementary Information for

Ton Motor Conformational Switch and Peptidoglycan Role in Bacterial Nutrient Uptake

Maximilian Zinke[1], Maylis Lejeune[1], Ariel Mechaly[2], Benjamin Bardiaux[1], Ivo Gomperts Boneca[3], Philippe Delepelaire[4,5], Nadia Izadi-Pruneyre[1]

[1] Institut Pasteur, Université Paris Cité, CNRS UMR3528, Bacterial Transmembrane Systems Unit, F-75015 Paris, France, [2] Institut Pasteur, Université Paris Cité, CNRS UMR3528, Crystallography Platform, F-75015 Paris, France, [3] Institut Pasteur, Université Paris Cité, CNRS UMR6047, INSERM U1306, Unité de Biologie et génétique de la paroi bactérienne F-75015, Paris, France, [4] Laboratoire de Biologie Physico-Chimique des Protéines Membranaires, Université Paris Cité, UMR7099 CNRS, F-75005, Paris, France, [5] Institut de Biologie Physico-Chimique, F-75005, Paris, France. Correspondence and requests for materials should be addressed to N.I-P. (email: nadia.izadi@pasteur.fr).

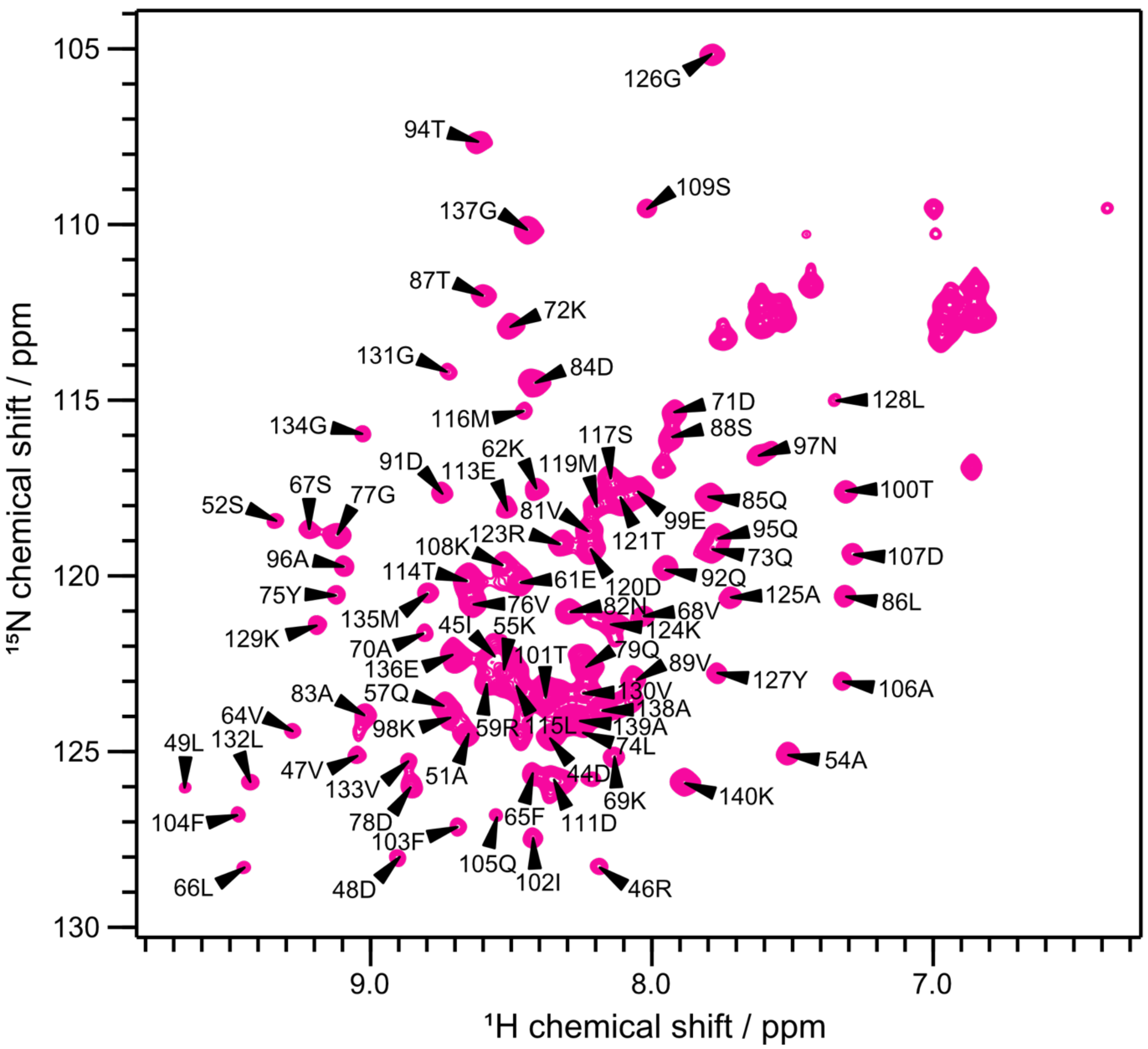

Supplementary Figure 1. 2D [^{1}H-^{15}N] SOFAST-HMQC spectrum of ^{15}N ExbD$^{Sm,\ peri}$ at 800 MHz external magnetic field, pH 7 and 293 K. Amide backbone resonance assignments are denoted by arrows, and their residue numbers as well as one-letter amino acid codes.

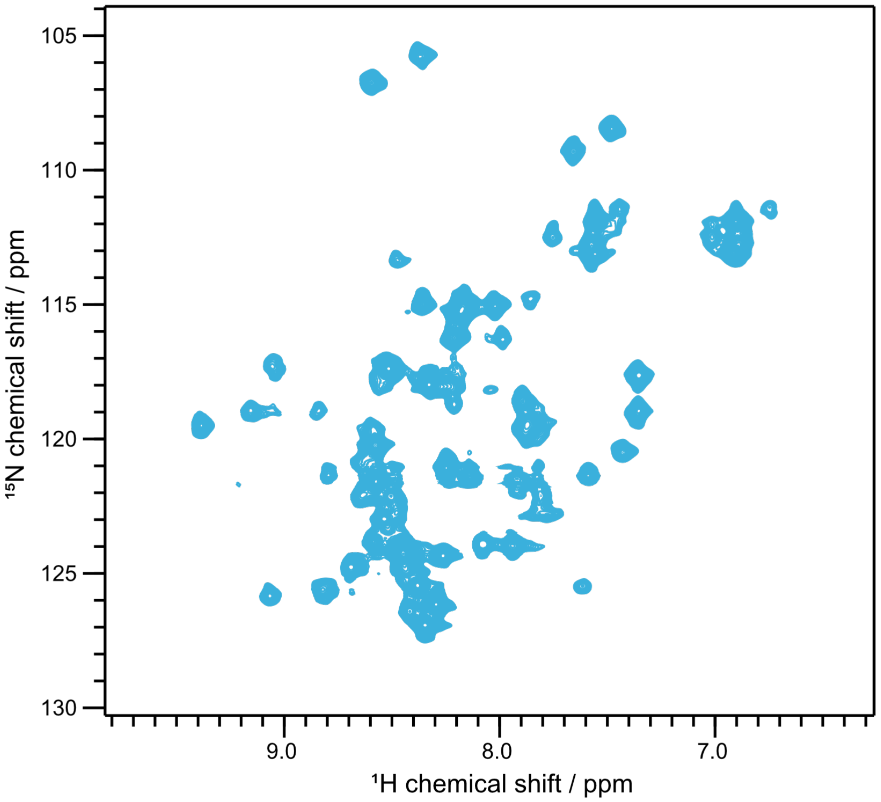

Supplementary Figure 2. 2D [^{1}H-^{15}N] SOFAST-HMQC spectrum of ^{15}N-labeled ExbD$^{Ec,\ peri}$ at 800 MHz external magnetic field, pH 7 and 293 K. Broadened peaks indicate exchange processes on the intermediate NMR timescale.

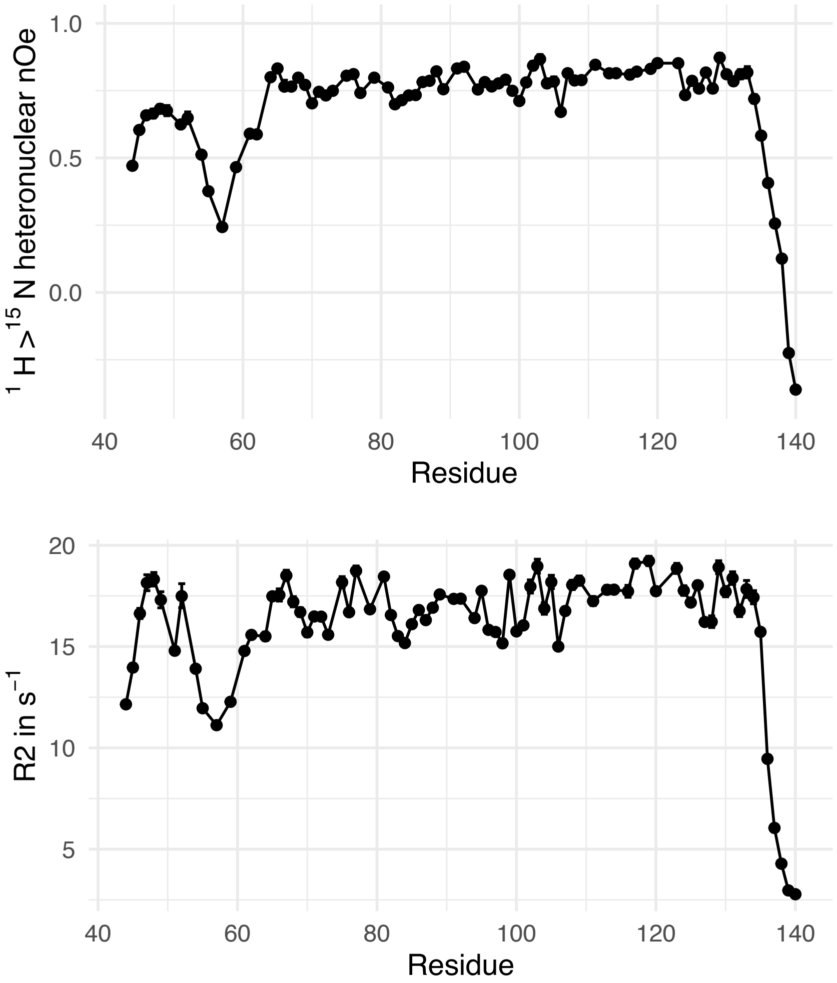

Supplementary Figure 3. $^{15}N[^1H]$ heteronuclear nuclear Overhauser effect measurements and R_2 relaxation rates of ^{15}N-labeled ExbD$^{Sm,\ peri}$ at 800 MHz external magnetic field. Source data are provided as a Source Data file. Data points represent mean values obtained from fitting NMR data, with error bars reflecting the standard deviation (SD) from Monte Carlo simulations performed for each residue, as detailed in the Methods.

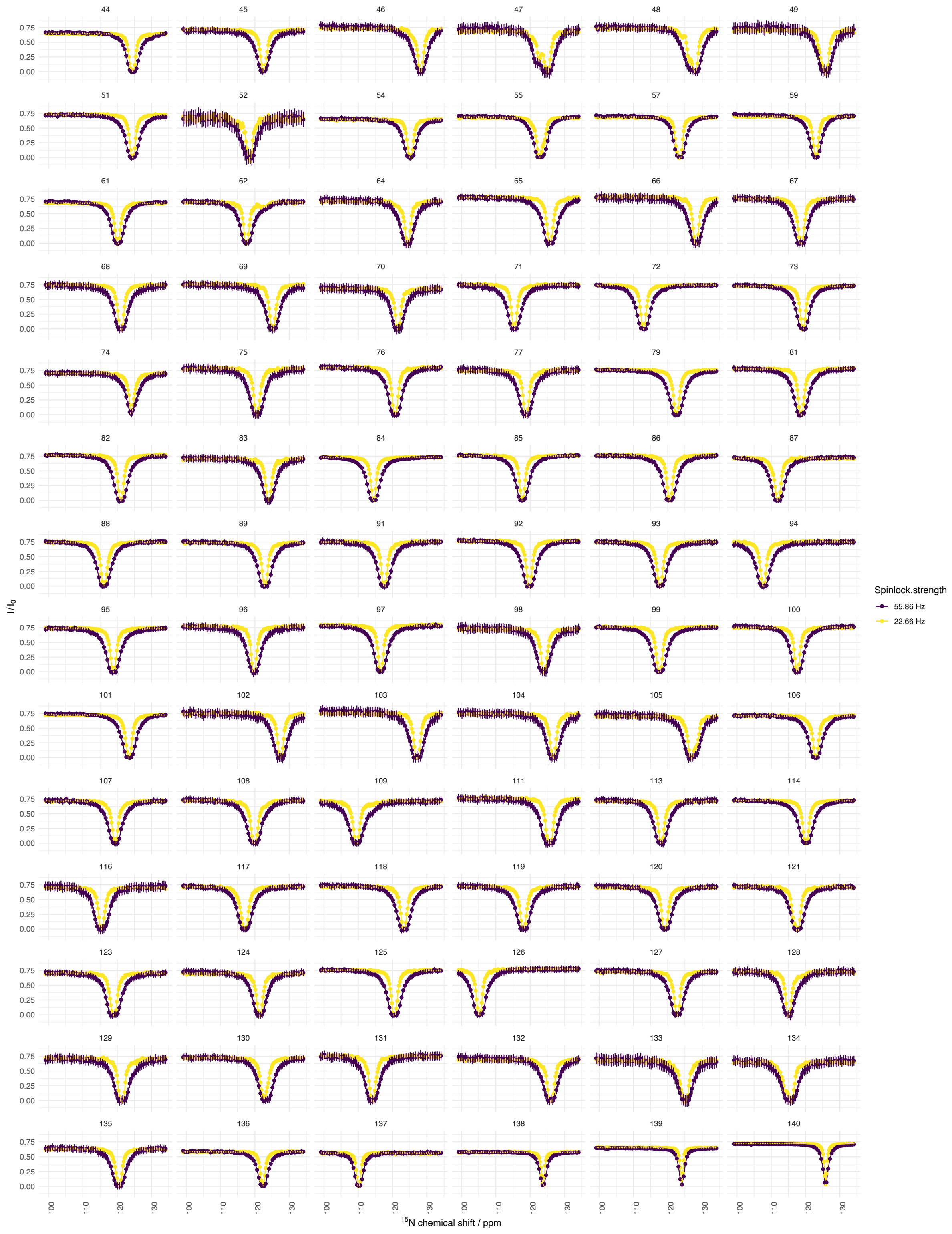

Supplementary Figure 4. Chemical exchange saturation transfer (CEST) profiles of ^{15}N-labeled ExbD$^{Sm,\ peri}$ at 55.9 Hz (purple) and 22.9 Hz (yellow) spinlock strength and 800 MHz external magnetic field. Missing residues are either due to peak overlap or to a low signal-to-noise. Source data are provided as a Source Data file. Peak intensities are plotted as mean values with error bars showing the SD, which quantifies the relative measurement error arising from noise in the NMR spectra.

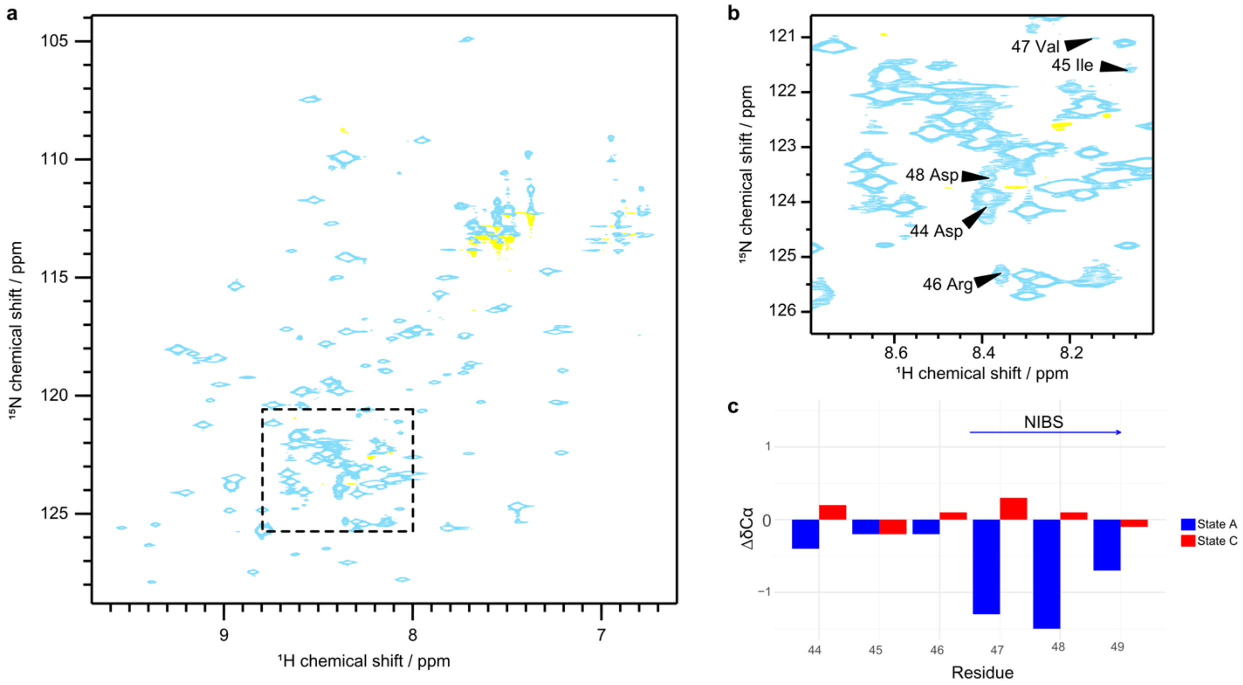

Supplementary Figure 5. Examination of the unfolded (open) State C of ExbD$^{Sm, peri}$. **a** High signal-to-noise 2D [^{1}H-^{15}N] TROSY spectrum of ^{15}N-labeled ExbD$^{Sm, peri}$, visualized at low contour levels, reveals a second set of peaks for the NIBS region. The region of interest is marked by a dashed rectangular outline. **b** Blowup of this region shows annotated peaks corresponding to the state C of the protein. The ^{15}N chemical shifts correspond to the values extracted from CEST experiments. Some residues are labeled and highlighted. **c** Secondary structure propensity of ExbD$^{Sm, peri}$ State A (blue) and State C (red). Negative secondary chemical shift values of Cα of State A correspond to a β-sheet (as represented by a blue arrow). In contrast, State C does not feature secondary structure.

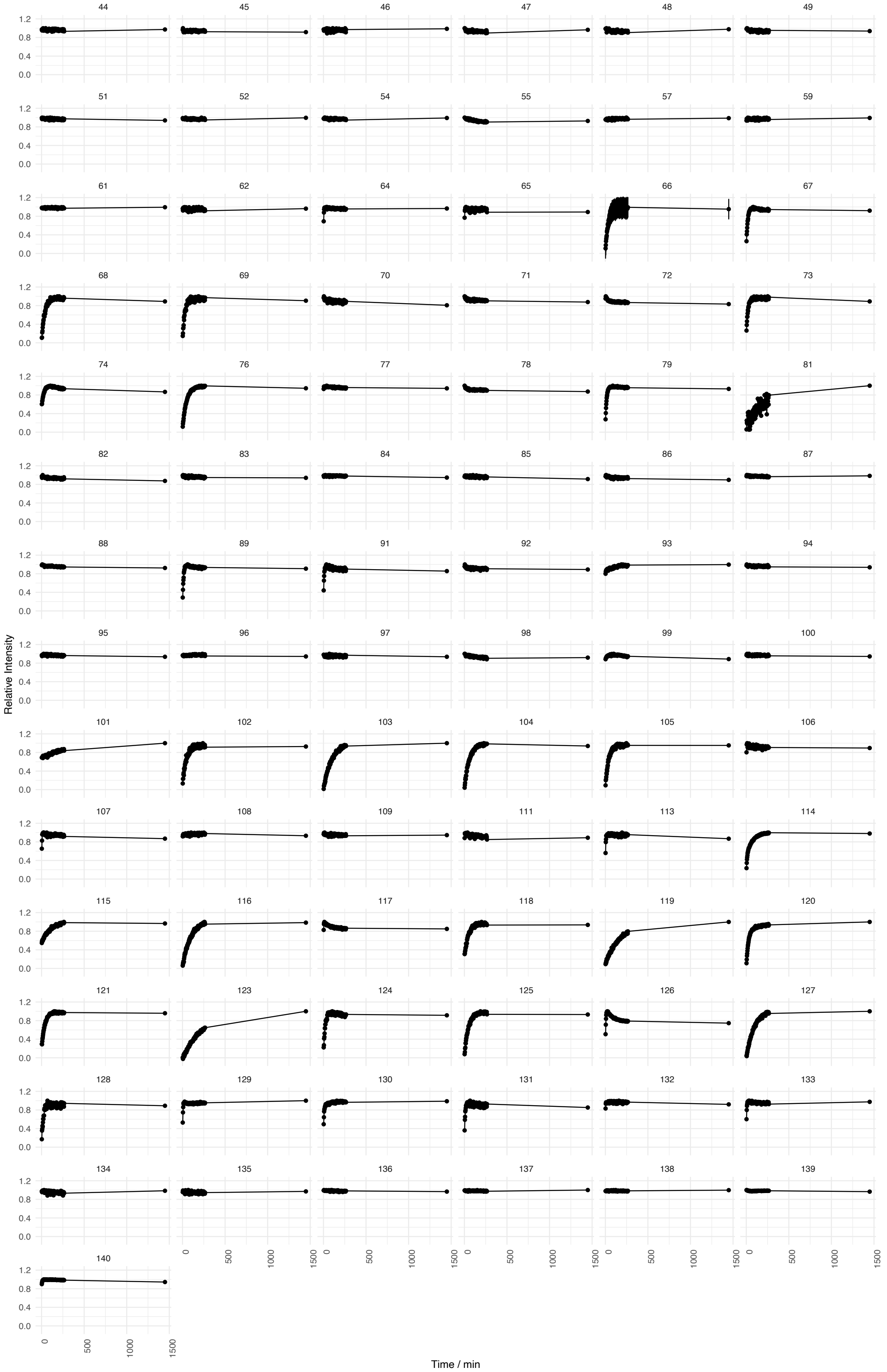

Supplementary Figure 6 Deuterium-hydrogen exchange (DHX) profiles of ^{15}N-labeled ExbD$^{Sm,\ peri}$ at 800 MHz external magnetic field. Missing residues are either the result of peak overlap or too low signal-to-noise. Amide group signal intensity variations are represented over time. The residue numbers are noted above each profile. Source data are provided as a Source Data file. Peak intensities are plotted as mean values with error bars showing the SD, which quantifies the relative measurement error arising from noise in the NMR spectra.

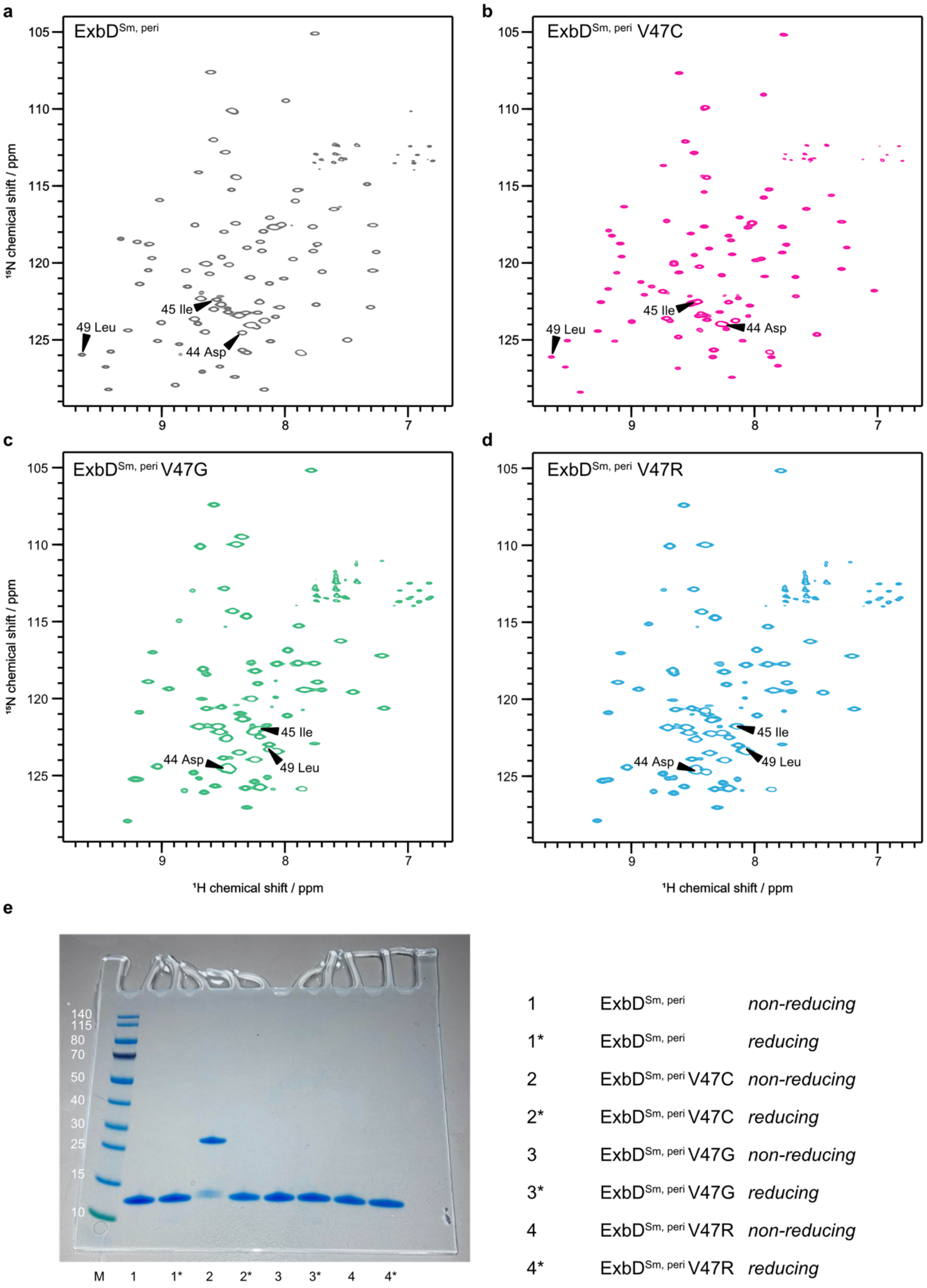

Supplementary Figure 7. a-d 2D [^{1}H-^{15}N] TROSY spectra of ^{15}N-labeled ExbD$^{Sm,\ peri}$ wt, V47C, V47G and V47R variants. While the V47C mutation induces minor chemical shift perturbations (CSPs) relative to the wild type, the V47G and V47R mutations result in substantial CSPs, especially for the NIBS residues. Moreover, these latter mutations lead to a distinctive peak concentration in the spectrum's central region, characteristic of a disordered structural state. **e**. SDS-PAGE of the same variants under non-reducing and reducing (50 mM TCEP) conditions show the formation of a disulfide bridge for the V47C mutant. Thus, while the V47C variant mirrors the wt structure – with only a substituted amino acid and an added disulfide bridge – the V47G and V47R variants retain the main fold but display the NIBS residues in a disordered state.

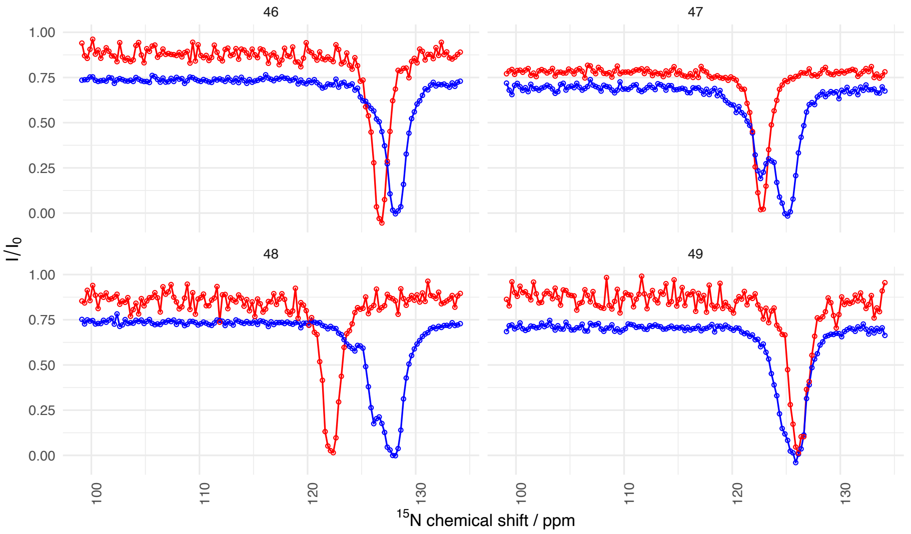

Supplementary Figure 8. The ^{15}N-Chemical Exchange Saturation Transfer (CEST) profiles showcase contrasting exchange dynamics between the NIBS of wild type (blue) and V47C mutant (red) of ^{15}N-labeled ExbD$^{Sm, peri}$. The data for residues 46 to 49 of the NIBS are shown. The residues from the wild type demonstrates a 3-state exchange, whereas those from the V47C mutant appear devoid of such exchange, showing significant alterations in the dynamics due to the disulfide bridge locking the NIBS in place. Source data are provided as a Source Data file. Peak intensities are plotted as mean values with error bars showing the SD, which quantifies the relative measurement error arising from noise in the NMR spectra.

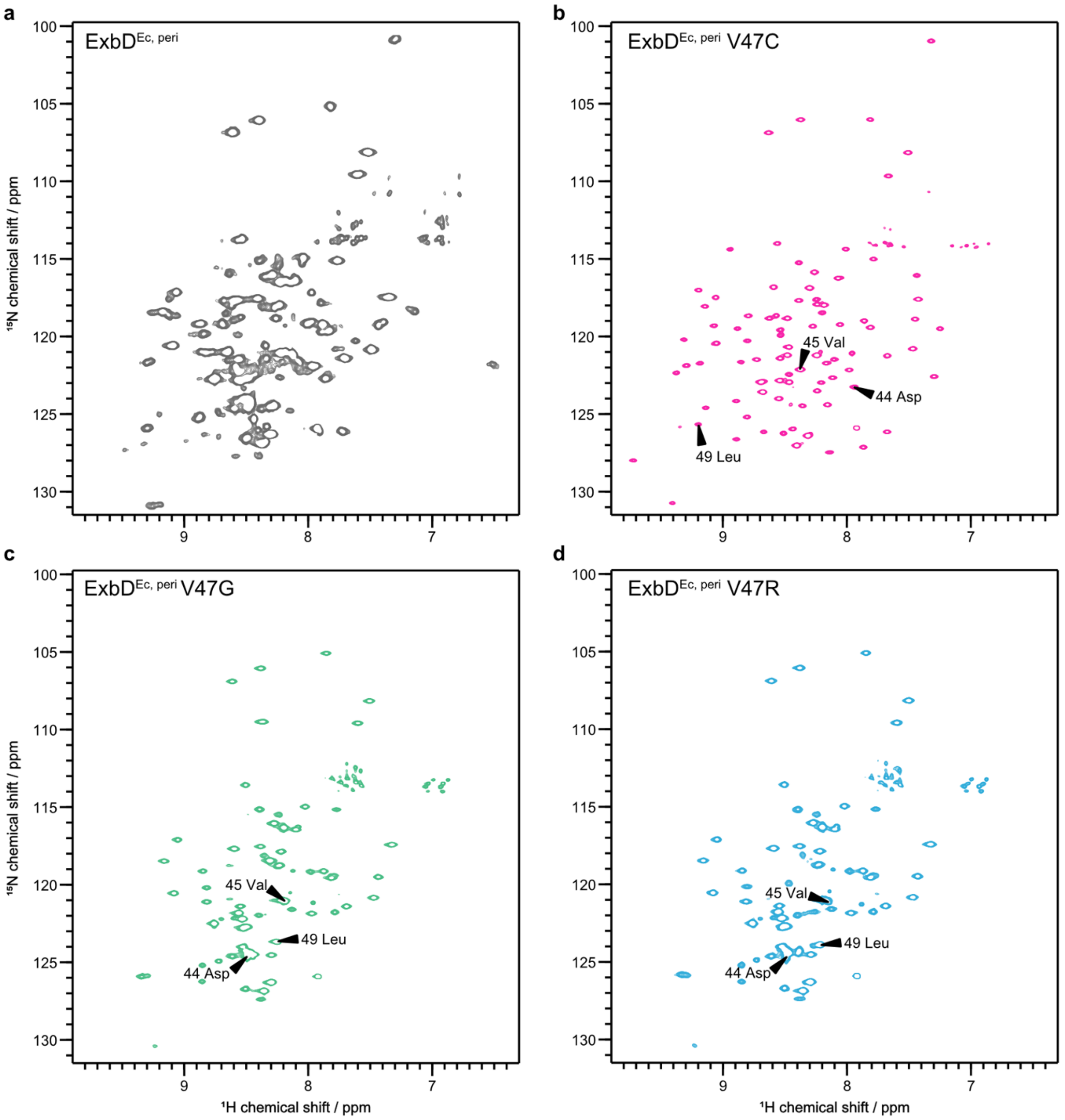

Supplementary Figure 9. 2D [^{1}H-^{15}N] TROSY spectra of ^{15}N-labeled ExbD$^{Ec, peri}$ wt, V47C, V47G and V47R variants. The spectrum of the wild type (a) is dominated by intermediate exchange at the NMR timescale (μs-ms), whereas the V47C mutation results in a high-quality spectrum (b) devoid of such exchange, exhibiting a quality comparable to the ExbD$^{Sm, peri}$ wt and V47C variants. On the other hand, the V47G (c) and V47R (d) mutations give rise to a distinctive concentration of peaks within the disordered region of the spectrum, similar to their ExbD$^{Sm, peri}$ counterparts.

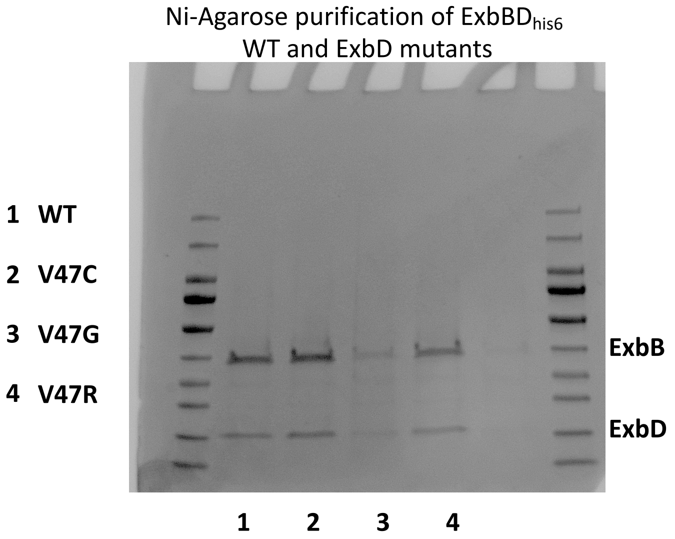

Supplementary Figure 10. SDS-PAGE after purification of the ExbB-ExbD complex from the membrane fraction. The similar quantities of the ExbB-ExbD complex containing the wt, V47C, V47R and V47G ExbD$^{Sm,\ peri}$ protein show that these mutants do not affect assembly and stability in the membrane.

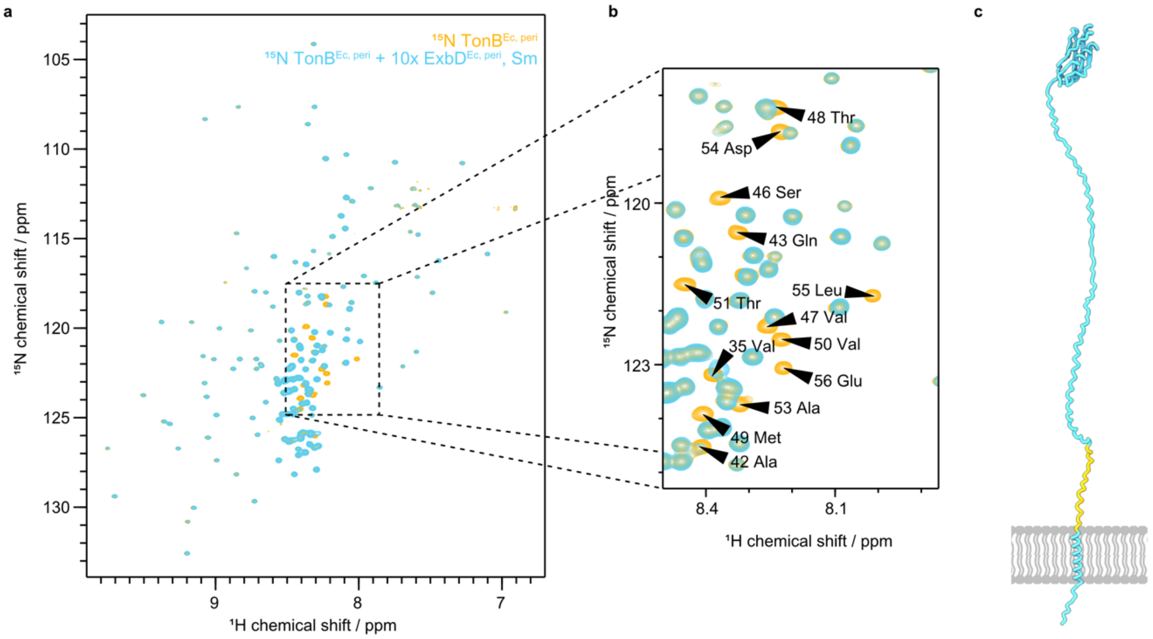

Supplementary Figure 11 TonB$^{Ec, peri}$ interacts with ExbD$^{Ec, peri}$. **a,b** 2D [1H-^{15}N] TROSY spectra of ^{15}N-labeled TonB$^{Ec, peri}$ without (orange) and with (blue) ExbD$^{Ec, peri}$. The presence of ExbD leads to the disappearance (broadening) of the residues 35-56 of TonB indicating binding of this region to ExbD. **c** These interacting residues (orange) are located in an intrinsically disordered region (IDR) of TonB (blue) close to the inner membrane inserted N-terminal α-helix.

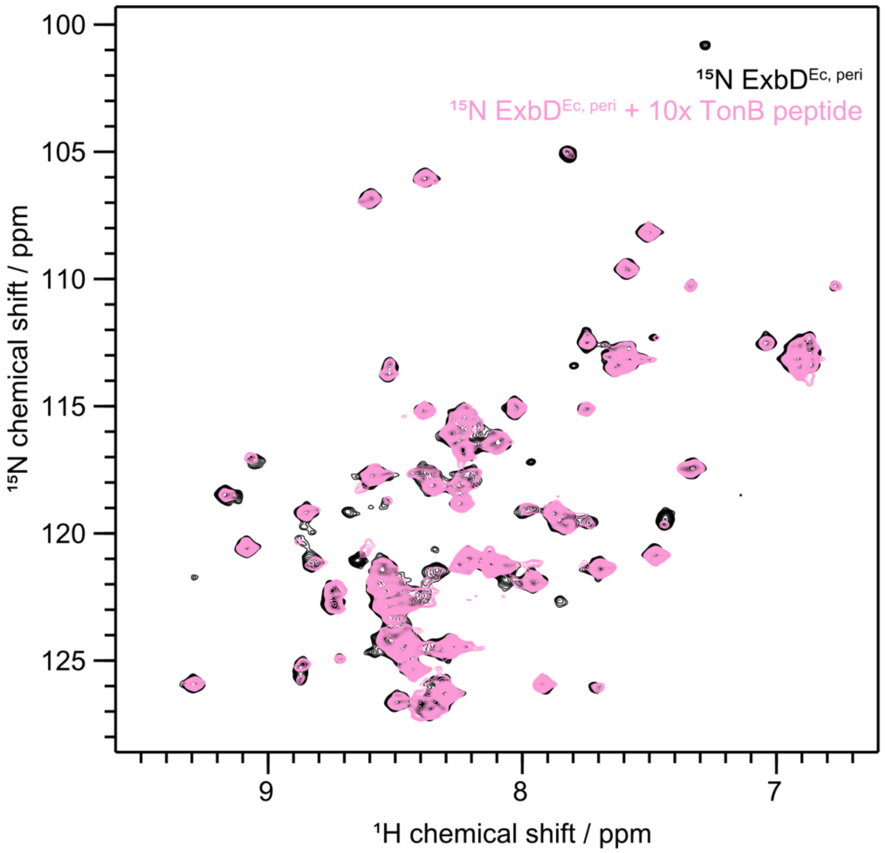

Supplementary Figure 12 2D [^{1}H-^{15}N] SOFAST-HMQC of ^{15}N-labeled ExbD$^{Ec, peri}$ without (black) and with (pink) the TonB peptide (that recalls the sequence of the binding region on the TonB side). Binding of the peptide leads to signal intensity decrease indicating an interaction.

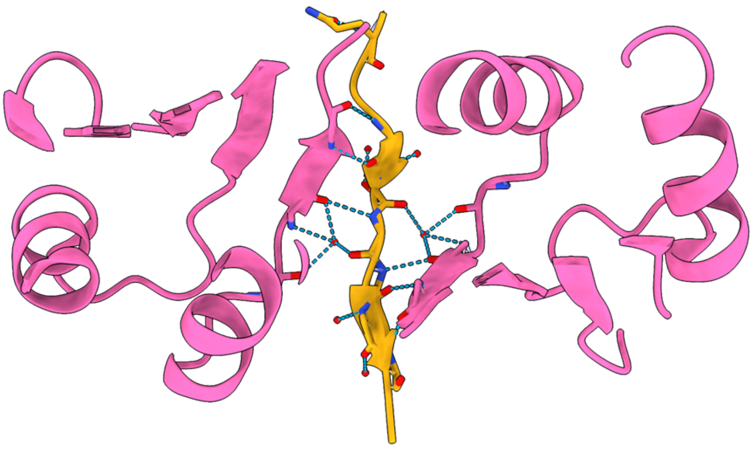

Supplementary Figure 13 Hydrogen bond (blue, dashed lines) interaction network between ExbD$^{Ec,\ \Delta NIBS}$ (pink) and the TonB peptide (orange). The TonB peptide binds in between the two ExbD protomers creating an intermolecular β-sheet. Hereby, it forms an antiparallel β-sheet with the β5-strand of one protomer and a parallel β-sheet with the β5-strand of the second protomer. In this representation for clarity, the topmost part of ExbD$^{Ec,\ \Delta NIBS}$ is not visible due to its location above the clipping plane.

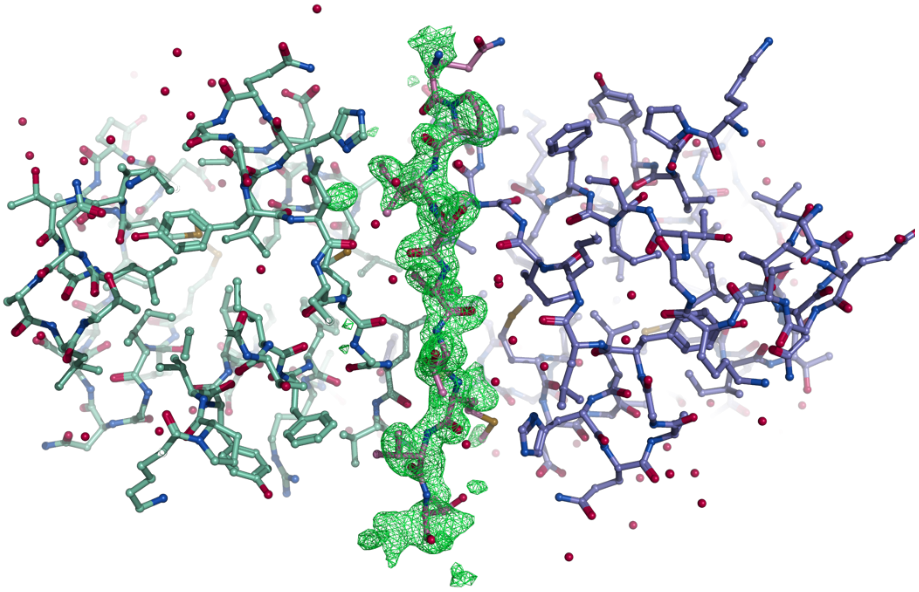

Supplementary Figure 14 The green mesh corresponds to a Polder omit map[1] (contoured at 3.0σ) calculated omitting the TonB peptide (in pink). ExbD dimer chains A and B are shown in sticks and colored green and violet, respectively. Water molecules are shown as red spheres.

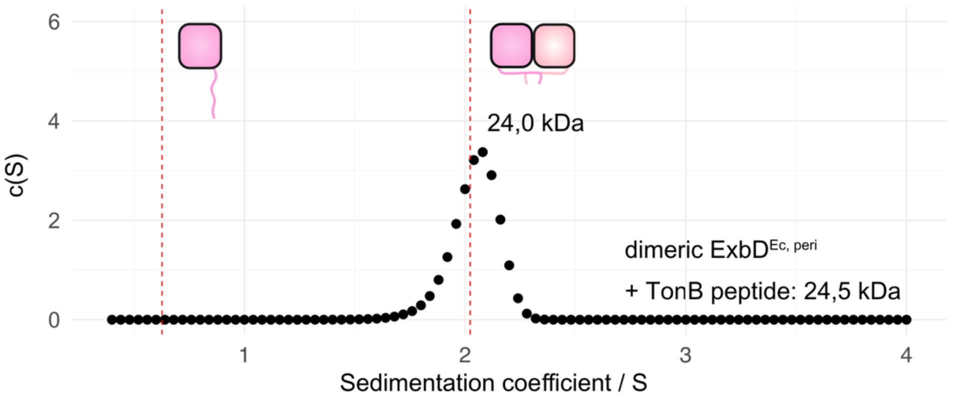

Supplementary Figure 15 Sedimentation coefficient distribution obtained from analytical ultracentrifugation of ExbD$^{Ec,\ peri}$ in the presence of the TonB peptide indicates that the ExbD-TonB complex maintains a dimeric organization of ExbD in solution. The red dashed lines represent estimated sedimentation coefficients for monomeric ExbD and dimeric ExbD as depicted by the cartoons (pink). The sedimentation coefficient-derived molecular weight of the complex is indicated above. Source data are provided as a Source Data file.

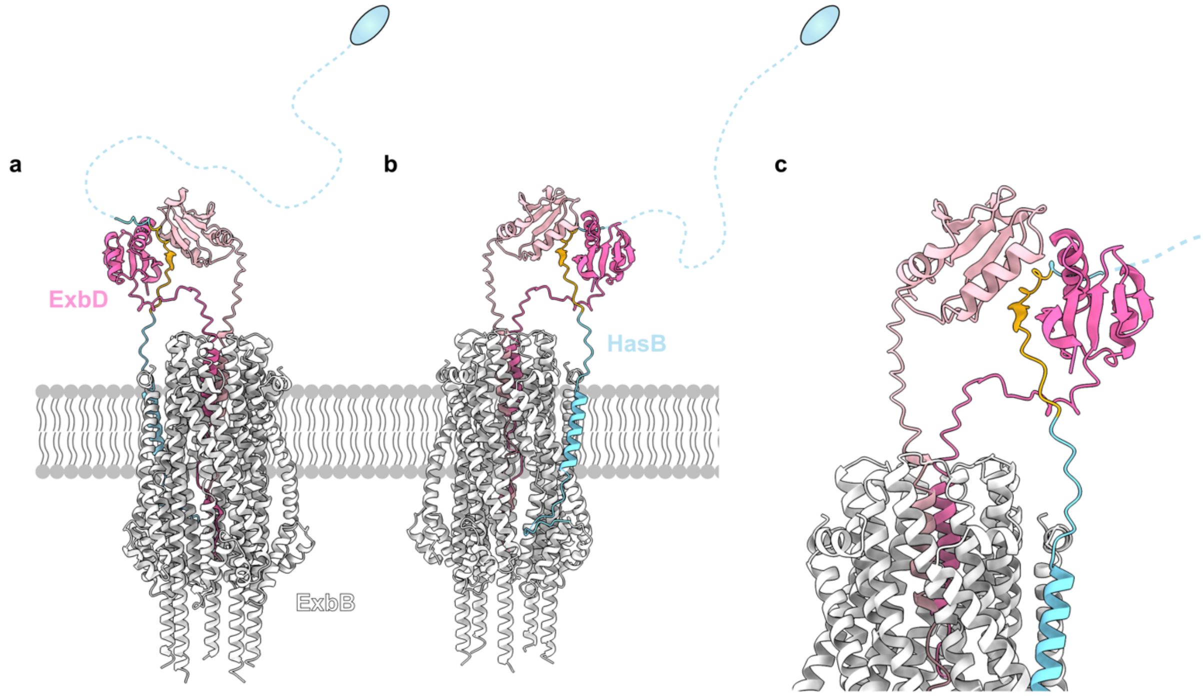

Supplementary Figure 16 AlphaFold2 model of the full ExbB-ExbD-HasB complex, generated using the crystal structure (PDB ID 8P9R, this work) as a template. **a, b** Side views of the model (model in **b** is rotated by 180° with respect to **a**) and **c** a zoom show that the dimensions for the ExbD-HasB interaction are feasible and do not present any spatial constraints as the unfolded NIBS residues act as hinges. The ExbD pentamer is represented in white, the ExbD dimer in pink and light pink, and HasB in turquoise. The region of interaction on the HasB side as identified by NMR spectroscopy is colored in orange. For simplicity, the C-terminal IDR and globular domain of HasB (68-263) are represented by a dashed line and a turquoise ellipse, respectively.

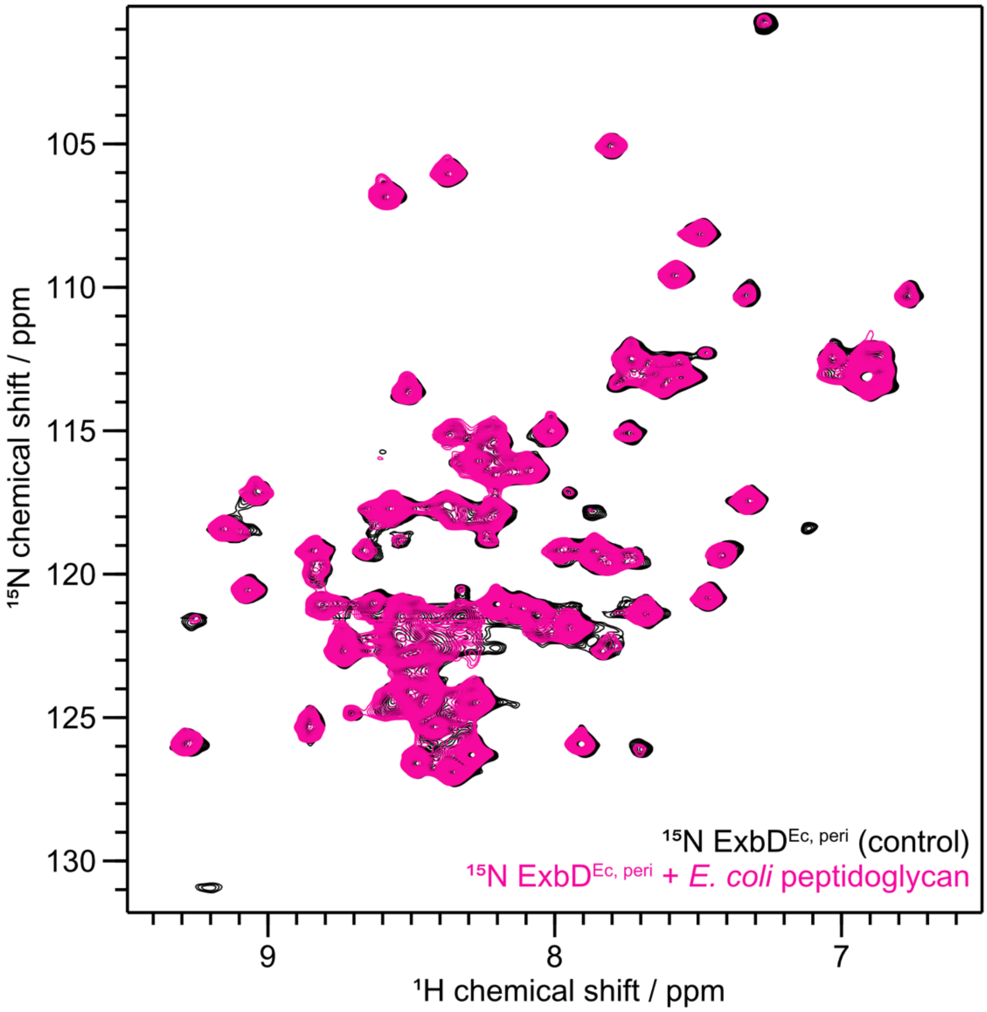

Supplementary Figure 17 2D [^{1}H-^{15}N] SOFAST-HMQC spectra of ^{15}N-labeled ExbD$^{Ec, peri}$ without (black) and with *E. coli* peptidoglycan (pink). The interaction of ExbD$^{Ec, peri}$ and peptidoglycan leads to the disappearance (broadening) of peaks.

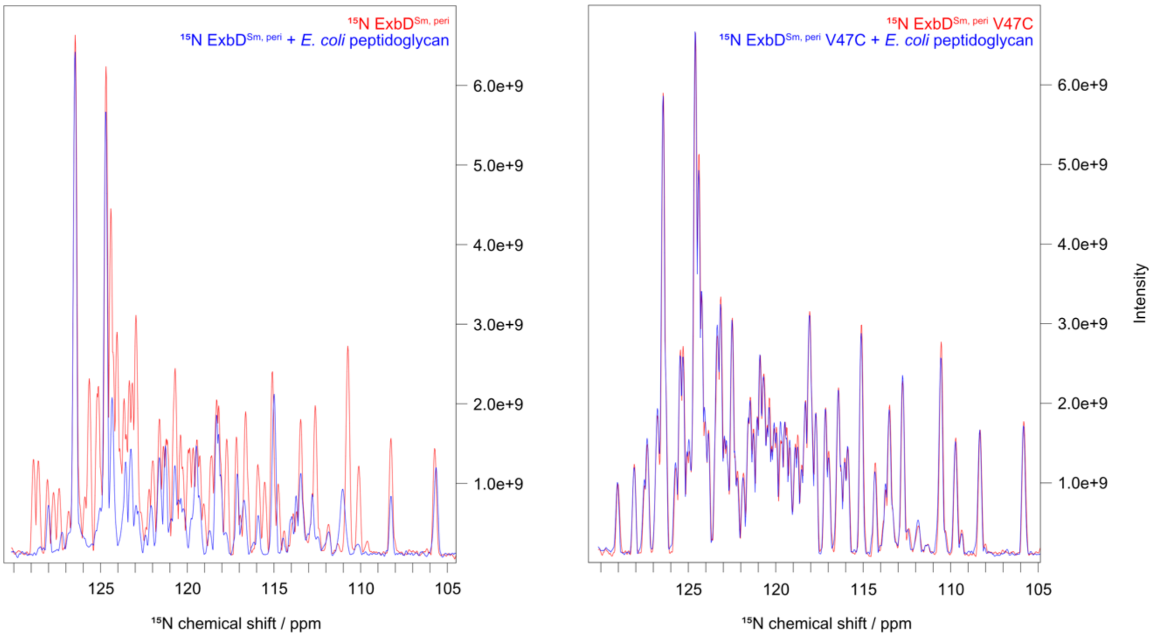

Supplementary Figure 18 Projections of 2D [^{1}H-^{15}N] TROSY spectra of ^{15}N-labeled ExbD$^{Sm, peri}$ wt (left) and V47C (right) in the absence (red) and presence (blue) of *E. coli* peptidoglycan sacculi. The interaction of ExbD$^{Sm, peri}$ wt and peptidoglycan leads to the disappearance (broadening) of peaks. Notably, ExbD$^{Sm, peri}$ V47C does not interact with peptidoglycan.

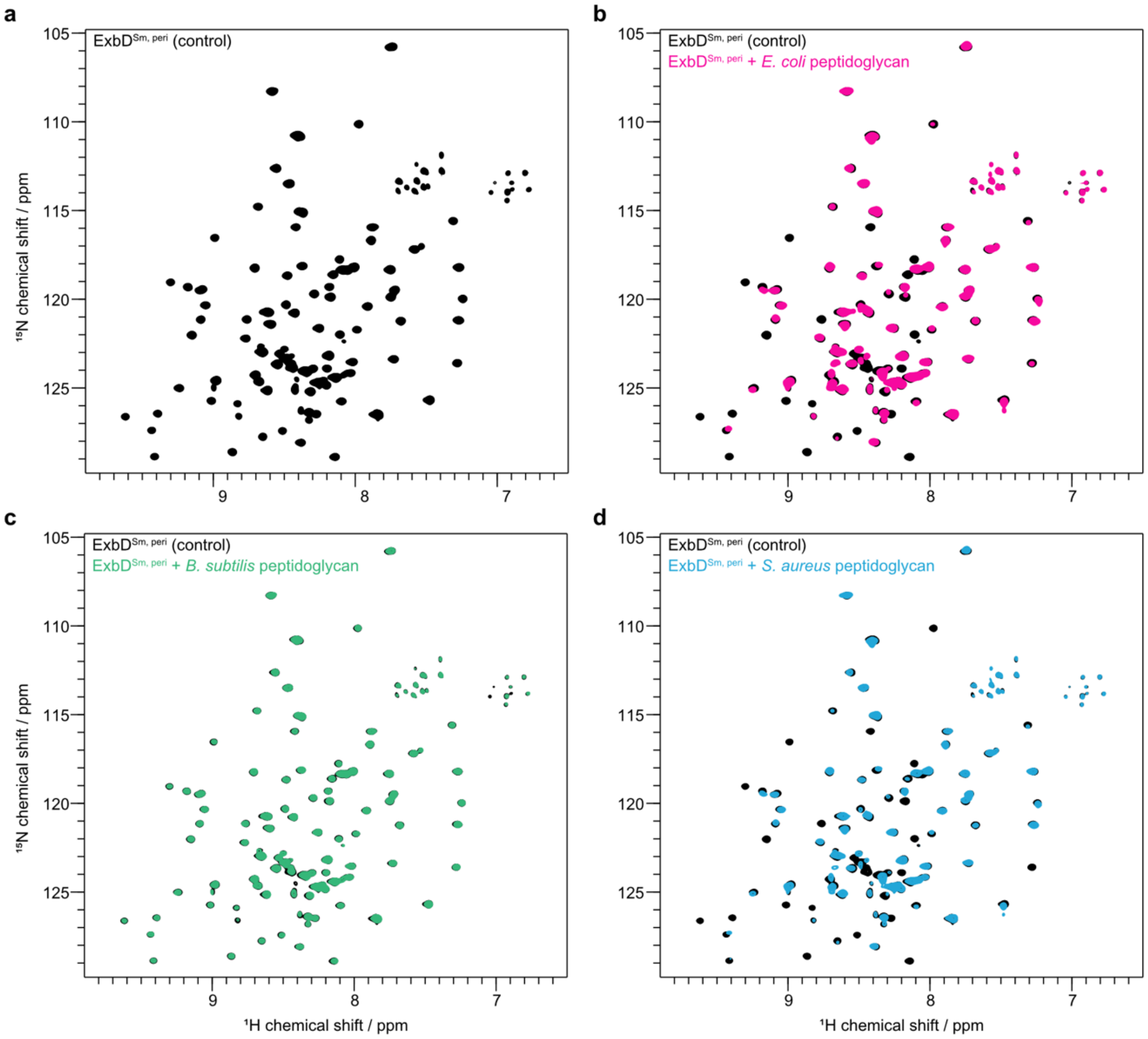

Supplementary Figure 19 2D [^{1}H-^{15}N] TROSY spectra of ^{15}N-labeled ExbD$^{Sm, peri}$ without (**a**, black) and with peptidoglycans (**b**, pink: *E. coli*; **c**, green: *B. subtilis*; **d**, blue: *S. aureus*). The interaction of ExbD$^{Sm, peri}$ and peptidoglycan (in b, and d) leads to the disappearance (broadening) of peaks. Notably, *B. subtilis* peptidoglycan (green) does not interact with ExbD$^{Sm, peri}$.

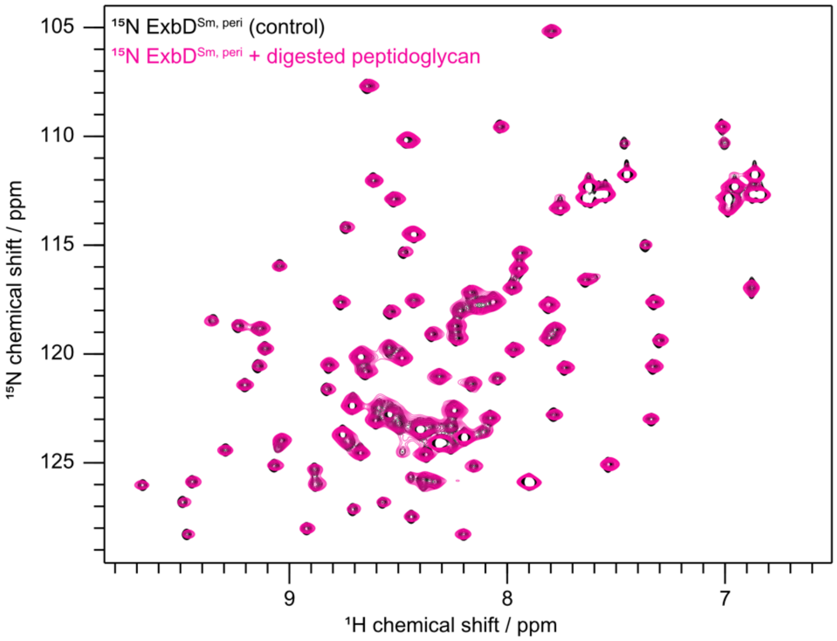

Supplementary Figure 20 2D [^{1}H-^{15}N] SOFAST-HMQC spectra of ^{15}N-labeled ExbD$^{Sm, peri}$ without (black) and with mutanolysin-digested, i.e. glycosidically-digested, *E. coli* peptidoglycan (pink). The spectral profiles between the two conditions exhibit no discernible changes, suggesting an absence of interaction. This reveals that an interaction between ExbD$^{Sm, peri}$ and peptidoglycan necessitates extended glycan stems on the side of peptidoglycan. Such stems are reduced to short saccharides in the mutanolysin-digested peptidoglycan, hence disabling the interaction.

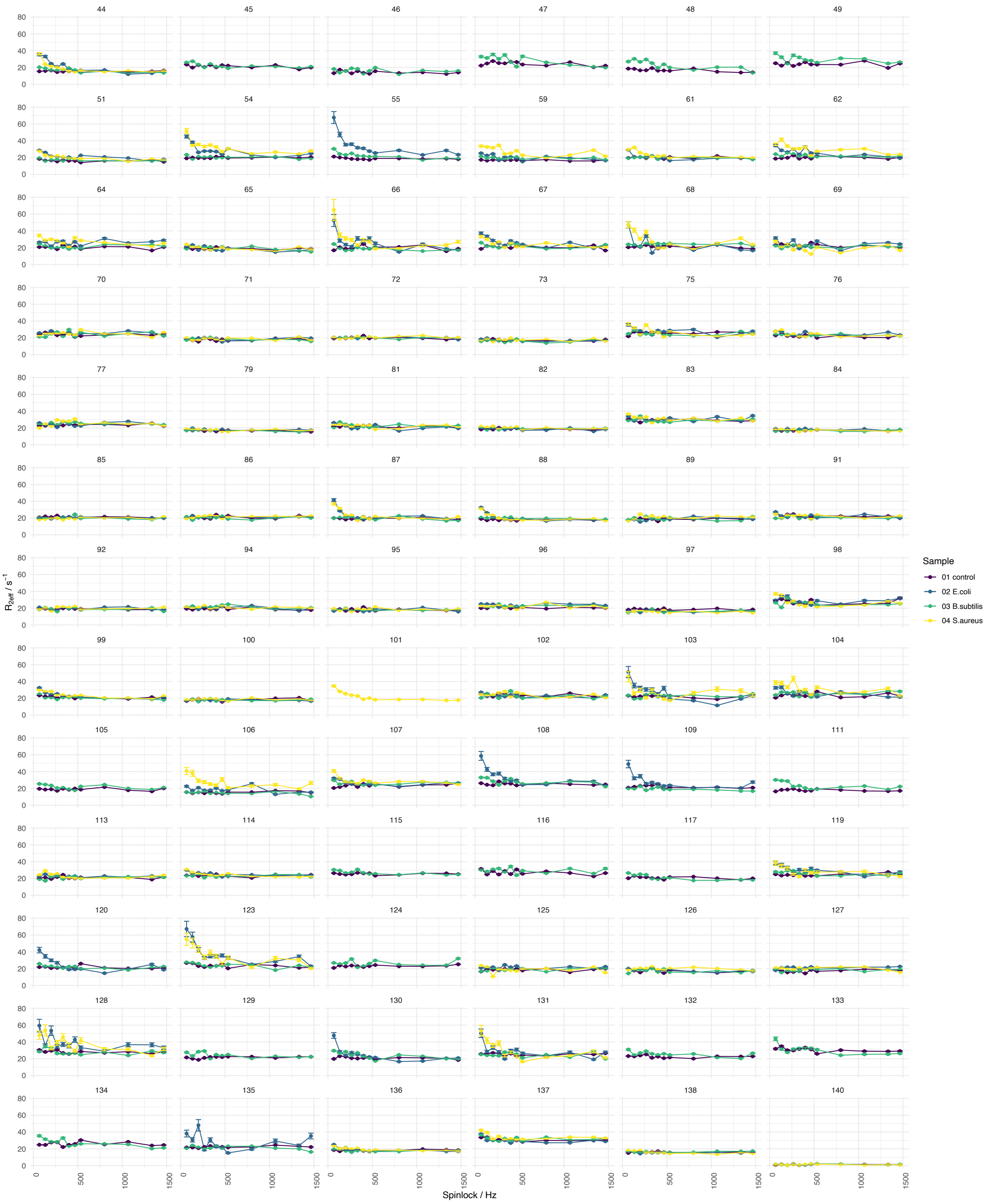

Supplementary Figure 21. CPMG relaxation dispersion profiles of ^{15}N-labeled ExbD$^{Sm,\ peri}$ with different peptidoglycan species. (Purple: control, blue: *E. coli* peptidoglycan, green: *B. subtilis* peptidoglycan, yellow: *S. aureus* peptidoglycan) at 600 MHz external magnetic field. Missing residues are either the result of peak overlap or too low signal-to-noise. Source data are provided as a Source Data file. Peak intensities are plotted as mean values with error bars showing the SD, which quantifies the relative measurement error arising from noise in the NMR spectra.

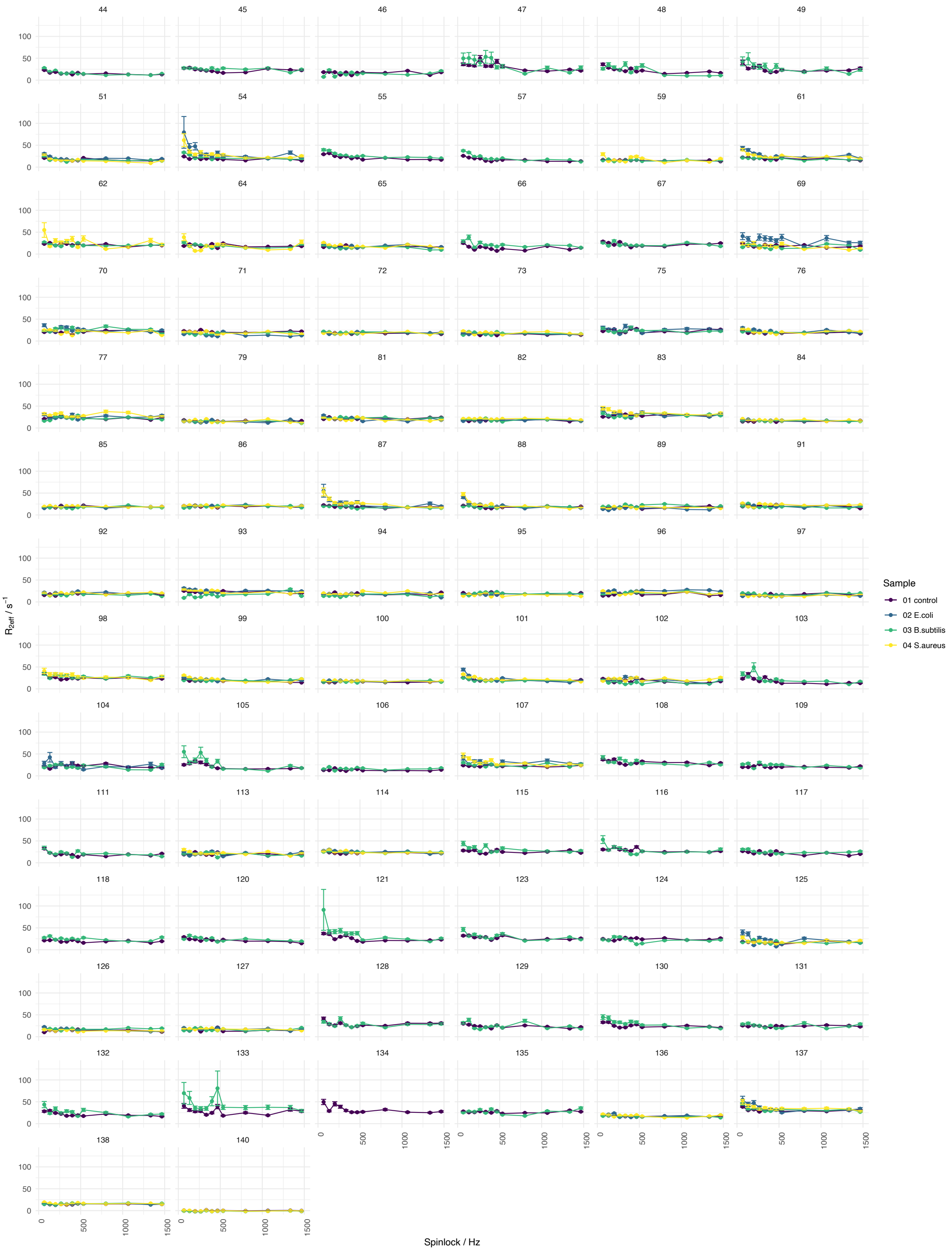

Supplementary Figure 22. CPMG relaxation dispersion profiles of ^{15}N-labeled ExbD$^{Sm,\ peri}$ with different peptidoglycan species. (Purple: control, blue: *E. coli* peptidoglycan, green: *B. subtilis* peptidoglycan, yellow: *S. aureus* peptidoglycan) at 800 MHz external magnetic field. Missing residues are either the result of peak overlap or too low signal-to-noise. Source data are provided as a Source Data file. Peak intensities are plotted as mean values with error bars showing the SD, which quantifies the relative measurement error arising from noise in the NMR spectra.

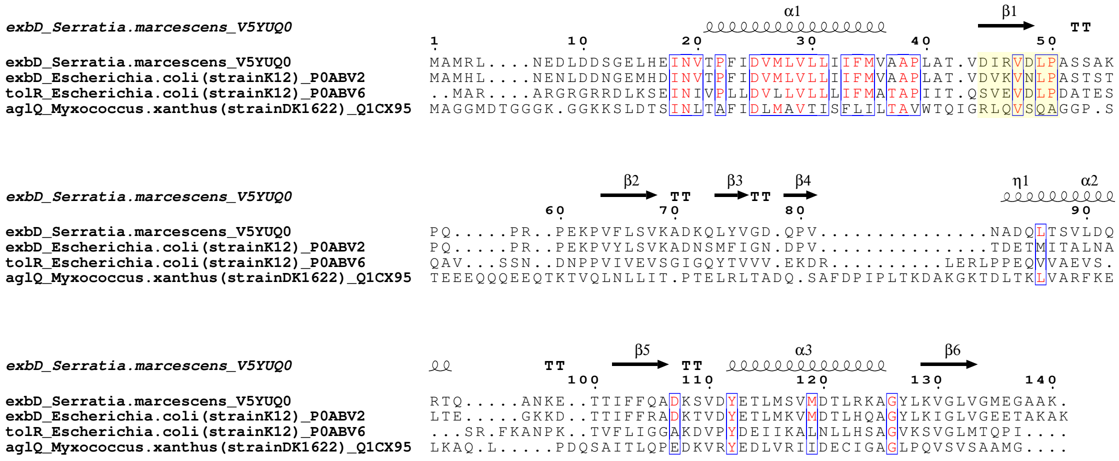

Supplementary Figure 23. Sequence alignment of ExbD homologues reveals that the NIBS (highlighted in yellow) is conserved across related systems. Homologous sequences of ExbD from *S. marcescens* and ExbD, TolR from *E. coli*, and AglQ from *Myxococcus xanthus* were identified using the ConSurf algorithm[2], employing standard parameters. The resulting 506 sequences were merged and aligned with Clustal Omega[3]. The sequence alignment was visualized by ENDscript[4]. For simplicity, only the input sequences are presented, though the similarity coloring scheme is based on all aligned sequences. The similarity score was computed by the percentage of strictly conserved residues per columns. The depicted secondary structure (top row) and the numbering are based on the *S. marcescens* ExbD sequence. Proteins are named by “gene-name_organism.name_uniprot-primary-accession-code”.

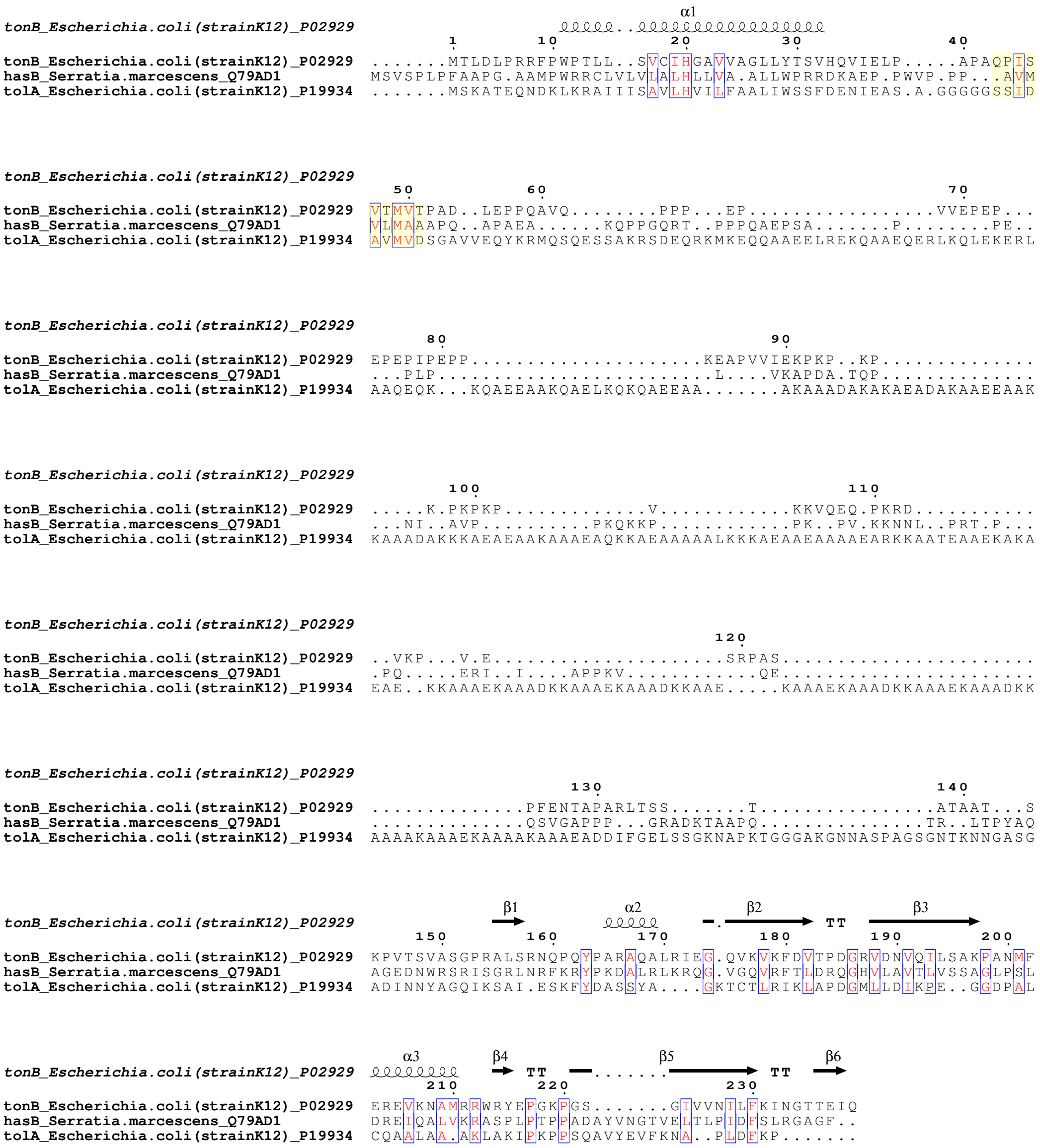

Supplementary Figure 24. Sequence alignment of TonB homologues reveals that the ExbD-binding motif (highlighted in yellow) is conserved among related systems. Homologous sequences of TonB, TolA from *E. coli*, and HasB from *S. marcescens* were identified using the ConSurf algorithm[2], employing standard parameters. The resulting 450 sequences were merged and aligned with Clustal Omega[3]. The sequence alignment was visualized by ENDscript[4]. For simplicity, only the input sequences are presented here, though the similarity coloring scheme is based on all aligned sequences. The similarity score was computed by the percentage of equivalent residues. The depicted secondary structure (top row) and the numbering are based on the *E. coli* TonB sequence. Proteins are named by "gene-name_organism.name_uniprot-primary-accession-code".

Supplementary Table 1. Protein/peptide sequences used in this study. The mutants V47C, V47R and V47G of $ExbD^{Sm,\ peri}$ and $ExbD^{Ec,\ peri}$ are not listed as they can be inferred from the wild type sequences.

Labeling alias	Comment	Sequence
$ExbD^{Sm,\ peri}$	Periplasmic domain of ExbD from *Serratia marcescens* (43-140)	SVDIRVDLPASSAKPQPRPEKPVFLSVKADKQLYVGDQPVNADQLTSVLD QRTQANKETTIFFQADKSVDYETLMSVMDTLRKAGYLKVGLVGMEGAAK
$ExbD^{Ec,\ peri}$	Periplasmic domain of ExbD from *Escherichia coli* (43-141)	SVDVKVNLPASTSTPQPRPEKPVYLSVKADNSMFIGNDPVTDETMITALN ALTEGKKDTTIFFRADKTVDYETLMKVMDTLHQAGYLKIGLVGEETAKAK
$ExbD^{Sm,\ \Delta NIBS}$	Periplasmic domain of ExbD from *Serratia marcescens* without NIBS (61-140)	SEKPVFLSVKADKQLYVGDQPVNADQLTSVLDQRTQANKETTIFFQADKS VDYETLMSVMDTLRKAGYLKVGLVGMEGAAK
$ExbD^{Ec,\ \Delta NIBS}$	Periplasmic domain of ExbD from *Escherichia coli* without NIBS (61-141)	SEKPVYLSVKADNSMFIGNDPVTDETMITALNALTEGKKDTTIFFRADKT VDYETLMKVMDTLHQAGYLKIGLVGEETAKAK
$HasB^{Sm,\ peri}$	Periplasmic domain of HasB from *Serratia marcescens* (37-263)	PRRDKAEPPWVPPPAVMVLMAAAPQAPAEAKQPPGQRTPPPQAEPSAPPE PLPLVKAPDATQPNIAVPPKQKKPPKPVKKNNLPRTPPQERIIAPPKVQE QSVGAPPPGRADKTAAPQTRLTPYAQAGEDNWRSRISGRLNRFKRYPKDA LRLKRQGVGQVRFTLDRQGHVLAVTLVSSAGLPSLDREIQALVKRASPLP TPPADAYVNGTVELTLPIDFSLRGAGF
$TonB^{Ec,\ peri}$	Periplasmic domain of TonB from *Escherichia coli* (34-239)	SQVIELPAPAQPISVTMVTPADLEPPQAVQPPPEPVVEPEPEPEPIPEPP KEAPVVIEKPKPKPKPKPKPVKKVQEQPKRDVKPVESRPASPFENTAPAR LTSSTATAATSKPVTSVASGPRALSRNQPQYPARAQALRIEGQVKVKFDV TPDGRVDNVQILSAKPANMFEREVKNAMRRWRYEPGKPGSGIVVNILFKI NGTTEIQ
HasB peptide	Region of HasB from *Serratia marcescens*	KKVPPPAVMVLMAAAPQAPAEKK
TonB peptide	Region of TonB from *Escherichia coli*	KKAQPISVTMVTPADLEPPQAKK

Supplementary Table 2. Protein samples for NMR spectroscopy and their purpose used in this study. Peptidoglycan is either *E. coli*, *B. subtilis* or *S. aureus* peptidoglycan.

**Labeling alias**	**Composition**	**Purpose**
$^{15}N,^{13}C$-ExbD$^{Sm, peri}$	1.5 mM $^{15}N,^{13}C$-ExbD$^{Sm, peri}$<br>50 mM sodium phosphate, pH 7.0<br>50 mM NaCl<br>5% D_2O	Assignment<br>^{15}N-NOESY-HSQC<br>T_1, T_2, heteronuclear nOe<br>CPMG relaxation dispersion<br>CEST
$^{15}N,^{13}C$-ExbD$^{Sm, peri}$ in D_2O	1.5 mM $^{15}N,^{13}C$-ExbD$^{Sm, peri}$<br>50 mM sodium phosphate, pH 7.0<br>50 mM NaCl<br>100% D_2O	^{13}C-NOESY-HSQC<br>DH-exchange
$^{2}H,^{15}N,^{13}C$-ExbD$^{Sm, peri}$	2 mM ^{2}H, $^{15}N,^{13}C$-ExbD$^{Sm, peri}$<br>50 mM sodium phosphate, pH 7.0<br>50 mM NaCl<br>5% D_2O	Hydrogen bond detection
$^{15}N,^{13}C$-ExbD$^{Ec, peri}$	750 μM $^{15}N,^{13}C$-ExbD$^{Ec, peri}$<br>50 mM sodium phosphate, pH 7.0<br>50 mM NaCl<br>5% D_2O	Assignment
50% $^{14}N,^{12}C$- & 50%$^{15}N,^{13}C$-ExbD$^{Sm, peri}$	1 mM $^{15}N,^{13}C$-ExbD$^{Sm, peri}$<br>1 mM $^{14}N,^{12}C$-ExbD$^{Sm, peri}$<br>50 mM sodium phosphate, pH 7.0<br>50 mM NaCl<br>5% D_2O	Intermolecular NOESY-HSQCs
$^{15}N,^{13}C$-ExbD$^{Sm, peri}$ V47C	0.54 mM $^{15}N,^{13}C$- ExbD$^{Sm, peri}$ V47C<br>50 mM sodium phosphate, pH 7.0<br>50 mM NaCl<br>5% D_2O	Assignment<br>Fingerprint
$^{15}N,^{13}C$-ExbD$^{Sm, peri}$ V47R	1.5 mM $^{15}N,^{13}C$- ExbD$^{Sm, peri}$ V47R<br>50 mM sodium phosphate, pH 7.0<br>50 mM NaCl<br>5% D_2O	Assignment<br>Fingerprint
$^{15}N,^{13}C$-ExbD$^{Sm, peri}$ V47G	1.2 mM $^{15}N,^{13}C$- ExbD$^{Sm, peri}$ V47G<br>50 mM sodium phosphate, pH 7.0<br>50 mM NaCl<br>5% D_2O	Assignment<br>Fingerprint
$^{15}N,^{13}C$-ExbD$^{Ec, peri}$ V47C	1.0 mM $^{15}N,^{13}C$- ExbD$^{Ec, peri}$ V47C<br>50 mM sodium phosphate, pH 7.0<br>50 mM NaCl<br>5% D_2O	Assignment<br>Fingerprint
$^{15}N,^{13}C$-ExbD$^{Ec, peri}$ V47R	1.0 mM $^{15}N,^{13}C$- ExbD$^{Ec, peri}$ V47C<br>50 mM sodium phosphate, pH 7.0<br>50 mM NaCl<br>5% D_2O	Assignment<br>Fingerprint
$^{15}N,^{13}C$-ExbD$^{Ec, peri}$ V47G	1.4 mM $^{15}N,^{13}C$- ExbD$^{Ec, peri}$ V47C<br>50 mM sodium phosphate, pH 7.0<br>50 mM NaCl<br>5% D_2O	Assignment<br>Fingerprint
$^{15}N,^{13}C$-ExbD$^{Sm, peri}$ + HasB peptide	50 μM $^{15}N,^{13}C$- ExbD$^{Sm, peri}$<br>(500 μM HasB peptide)<br>50 mM sodium phosphate, pH 7.0<br>50 mM NaCl<br>5% D_2O	Fingerprint (without and with peptide)<br>T2 relaxation
$^{15}N,^{13}C$-ExbD$^{Ec, peri}$ + TonB peptide	50 μM $^{15}N,^{13}C$-ExbD$^{Ec, peri}$<br>(500 μM TonB peptide)<br>50 mM sodium phosphate, pH 7.0<br>50 mM NaCl<br>5% D_2O	Fingerprint (without and with peptide)

$^{15}N,^{13}C$-ExbD$^{Sm, peri}$ + peptidoglycan	100 µM $^{15}N,^{13}C$-ExbD$^{Sm, peri}$<br>(0.5 mg peptidoglycan)<br>50 mM sodium phosphate, pH 7.0<br>50 mM NaCl<br>5% D_2O	Fingerprint (without and with peptidoglycan)<br>CPMG relaxation dispersion
$^{15}N,^{13}C$-ExbD$^{Sm, peri}$ V47C + peptidoglycan	100 µM $^{15}N,^{13}C$-ExbD$^{Sm, peri}$ V47C<br>(0.5 mg peptidoglycan)<br>50 mM sodium phosphate, pH 7.0<br>50 mM NaCl<br>5% D_2O	Fingerprint (without and with peptidoglycan)
$^{15}N,^{13}C$-ExbD$^{Sm, peri}$ + digested peptidoglycan	200 µM $^{15}N,^{13}C$-ExbD$^{Sm, peri}$<br>2 mM digested peptidoglycan<br>50 mM sodium phosphate, pH 7.0<br>50 mM NaCl<br>5% D_2O	Fingerprint
$^{15}N,^{13}C$-HasB$^{Sm, peri}$	50 µM $^{15}N,^{13}C$-HasB$^{Sm, peri}$<br>(500 µM $^{14}N,^{12}C$-ExbD$^{Sm, peri}$)<br>50 mM sodium phosphate, pH 7.0<br>50 mM NaCl<br>5% D_2O	Fingerprint (without and with ExbD_Sm_CTD43)<br>Assignment
$^{15}N,^{13}C$-TonB$^{Ec, peri}$	50 µM $^{15}N,^{13}C$-TonB$^{Ec, peri}$<br>(500 µM $^{14}N,^{12}C$-ExbD$^{Ec, peri}$)<br>50 mM sodium phosphate, pH 7.0<br>50 mM NaCl<br>5% D_2O	Fingerprint (without and with ExbD_Ec_CTD43)
$^{15}N,^{13}C$-TonB$^{Ec, peri}$ (2)	650 µM $^{15}N,^{13}C$-TonB$^{Ec, peri}$<br>50 mM sodium phosphate, pH 7.0<br>50 mM NaCl<br>5% D_2O	Assignment

Supplementary Table 3. NMR structure and restraints statistics of the final consensus calculation for ExbD$^{Sm, peri}$.

**Number of NMR restraints (restraints (per monomer)**	
Short-range (|i-j|≤1)	1603
Medium-range (1<|i-j|<5)	737
Long-range (|i-j|≥5)	1155
Ambiguous	732
Inter-molecular	318
Total	4545
Hydrogen bond restraints (intra | inter)	2 | 0
Dihedral angle restraints (phi | psi)	80 | 80
**Restraint violation**	
NMR distance restraints	
RMS of violations (Å)	0.05 ± 0.01
Number of violations >0.3 Å	71.45 ± 8.20
Number of violations >0.1 Å	134.75 ± 9.66
Hydrogen bond restraints	
RMS of violations (Å)	0.00 ± 0.00
Number of violations >0.1 Å	0.00 ± 0.00
Dihedral angle restraints	
RMS of violations (°)	0.21 ± 0.032
Number of violations >5°	0.00 ± 0.000
**Deviations from ideal geometry**	
RMS for bond length (Å)	0.004 ± 0.0001
RMS for bond angles (°)	0.53 ± 0.009
RMS for impropers (°)	1.5 ± 0.064
**Ramachandran plot statistics (%)**	
Most favored regions	85.90 ± 1.91
Allowed regions	12.90 ± 2.21
Generously allowed regions	0.60 ± 0.63
Disallowed region	0.60 ± 0.63
**RMSD from average structure** (Å)	
All residues	
Backbone atoms	0.41 ± 0.04
Heavy atoms	0.67 ± 0.06
Ordered residues (44:51,54:136)	
Backbone atoms	0.33 ± 0.04
Ordered residues	0.64 ± 0.06

Supplementary Table 4. Parameters for the fitted three-state exchange of $ExbD^{Sm, peri}$. Fitted ^{15}N chemical shifts are compared to the ^{15}N chemical shifts of the new peaks upon addition of HasB peptide to $ExbD^{Sm, peri}$. Comparing the exchange rates to the chemical shift difference, AB can be classified as intermediate, AC as slow and BC also as slow exchange on the NMR timescale.

	**Chemical shift / ppm**			
	**From CEST data**			
**Residue**	**State A**	**State B**	**State C**	**Upon peptide addition**
44 Asp	124.62	123.57 ± 0.01	125.13 ± 0.08	125.57
45 Ile	122.45	123.32 ± 0.02	121.59 ± 0.11	121.81
46 Arg	128.29	128.62 ± 0.06	126.56 ± 0.11	125.56
47 Val	125.13	122.47 ± 0.03	120.99 ± 0.12	121.17
48 Asp	128.01	126.04 ± 0.02	124.18 ± 0.07	123.92
49 Leu	126.02	124.58 ± 0.03	123.81 ± 0.10	123.69
55 Lys	122.74	124.26 ± 0.02	121.42 ± 0.07	122.22

**Global exchange parameters**				
$K_{ex}(AB)$	$K_{ex}(AC)$	$K_{ex}(BC)$	P_B	P_C
221.9 ± 8.4 Hz	50.5 ± 7.6 Hz	0.0008 ± 0.07 Hz	3.56 ± 0.0006 %	1.6 ± 0.002 %

Supplementary Table 5. Crystallography data collection and refinement statistics.

**Parameters**	**ExbD$^{Ec, \Delta NIBS}$ + TonB peptide**
Beamline	Proxima 1
Resolution range (Å)	39.07-1.52 (1.61-1.52)
Space group	P4(1)
Unit cell (Å / °)	49.3 49.3 63.9<br>90.0 90.0 90.0
Total reflections	618467 (97962)
Unique reflections	46421 (7485)
Multiplicity	13.3
Completeness (%)	99.9 (99.9)
Mean I/sigma(I)	21.45 (2.45)
Wilson B-factor	35.0
R-meas	0.061 (0.816)
R-pim	0.058 (0.784)
CC1/2	0.999 (0.897)
Reflections used in refinement	23622
Reflections used for R-free	1181
R-work	0.2101 (0.3263)
R-free	0.2376 (0.3696)
Number of non-hydrogen atoms	1293
Macromolecules	1203
Solvent	90
Protein residues	156
RMS (bonds) (Å)	0.007
RMS (angles) (°)	1.34
Ramachandran plot	
Favored (%)	100.00
Allowed (%)	0.00
Outliers (%)	0.00
Rotamer Outliers (%)	0.74
Clashscore	6.19
B-factors	
Average	33.59
Macromolecules	33.17
Solvent	39.23

Supplementary Table 6. Plasmids used in this study.

Name	Plasmid Vector	Protein	Source/Reference
pET_ExbD_Sm_peri	pET-30a(+)	$ExbD^{Sm,\ peri}$	ProteoGenix, this study
pET_ExbD_Sm_peri_V47C	pET-30a(+)	$ExbD^{Sm,\ peri}$ V47C	ProteoGenix, this study
pET_ExbD_Sm_peri_V47R	pET-30a(+)	$ExbD^{Sm,\ peri}$ V47R	ProteoGenix, this study
pET_ExbD_Sm_peri_V47G	pET-30a(+)	$ExbD^{Sm,\ peri}$ V47G	ProteoGenix, this study
pET_ExbD_Ec_peri	pET-30a(+)	$ExbD^{Ec,\ peri}$	ProteoGenix, this study
pET_ExbD_Ec_peri_V47C	pET-30a(+)	$ExbD^{Ec,\ peri}$ V47C	ProteoGenix, this study
pET_ExbD_Ec_peri_V47R	pET-30a(+)	$ExbD^{Ec,\ peri}$ V47R	ProteoGenix, this study
pET_ExbD_Ec_peri_V47G	pET-30a(+)	$ExbD^{Ec,\ peri}$ V47G	ProteoGenix, this study
pET_ExbD_Sm_ΔNIBS	pET-30a(+)	$ExbD^{Sm,\ \Delta NIBS}$	ProteoGenix, this study
pET_ExbD_Ec_ΔNIBS	pET-30a(+)	$ExbD^{Ec,\ \Delta NIBS}$	ProteoGenix, this study
pET_HasB_Sm_peri	pET-30a(+)	$HasB^{Sm,\ peri}$	ProteoGenix, this study
pET_TonB_Ec_peri	pET-30a(+)	$TonB^{Ec,\ peri}$	ProteoGenix, this study
pBAD24	pBAD24	none	Lab collection
pBAD24exbBDSm	pBAD24	$ExbB^{Sm}$, $ExbD^{Sm}$	Lab collection
pBAD24exbBDV47CSm	pBAD24	$ExbB^{Sm}$, $ExbD^{Sm}$ V47C	This study
pBAD24exbBDV47GSm	pBAD24	$ExbB^{Sm}$, $ExbD^{Sm}$ V47G	This study
pBAD24exbBDV47RSm	pBAD24	$ExbB^{Sm}$, $ExbD^{Sm}$ V47R	This study
pBAD24exbBDD111CSm	pBAD24	$ExbB^{Sm}$, $ExbD^{Sm}$ D111C	This study
pBAD24exbBDhis6Sm	pBAD24	$ExbB^{Sm}$, $ExbD^{Sm,\ his6}$	Lab collection
pBAD24exbBDV47Chis6Sm	pBAD24	$ExbB^{Sm}$, $ExbD^{Sm,\ his6}$ V47C	This study
pBAD24exbBDV47Ghis6Sm	pBAD24	$ExbB^{Sm}$, $ExbD^{Sm,\ his6}$ V47G	This study
pBAD24exbBDV47Rhis6Sm	pBAD24	$ExbB^{Sm}$, $ExbD^{Sm,\ his6}$ V47R	This study
pBAD24exbBDD111Chis6Sm	pBAD24	$ExbB^{Sm}$, $ExbD^{Sm,\ his6}$ D111C	This study

REFERENCES

1. Liebschner, D. *et al.* Polder maps: improving OMIT maps by excluding bulk solvent. *Acta Crystallogr. Sect. Struct. Biol.* **73**, 148–157 (2017).
2. Ashkenazy, H. *et al.* ConSurf 2016: an improved methodology to estimate and visualize evolutionary conservation in macromolecules. *Nucleic Acids Res.* **44**, W344–W350 (2016).
3. Sievers, F. *et al.* Fast, scalable generation of high-quality protein multiple sequence alignments using Clustal Omega. *Mol. Syst. Biol.* **7**, 539 (2011).
4. Robert, X. & Gouet, P. Deciphering key features in protein structures with the new ENDscript server. *Nucleic Acids Res.* **42**, W320–W324 (2014).
