# Supplementary material for: Ton motor conformational switch and peptidoglycan role in bacterial nutrient uptake
Source: Nat Commun. 2024 Jan 6;15:331. doi: 10.1038/s41467-023-44606-z (PMC10771500; doi:10.1038/s41467-023-44606-z)
Supplement: Supplementary file 3 — Reporting Summary [file 41467_2023_44606_MOESM3_ESM.pdf]

nature portfolio

Corresponding author(s): Nadia Izadi-Pruneyre

Last updated by author(s): Nov 23, 2023

Reporting Summary

Nature Portfolio wishes to improve the reproducibility of the work that we publish. This form provides structure for consistency and transparency in reporting. For further information on Nature Portfolio policies, see our Editorial Policies and the Editorial Policy Checklist.

Statistics

For all statistical analyses, confirm that the following items are present in the figure legend, table legend, main text, or Methods section.

n/a	Confirmed	
☐	☒	The exact sample size (n) for each experimental group/condition, given as a discrete number and unit of measurement
☐	☒	A statement on whether measurements were taken from distinct samples or whether the same sample was measured repeatedly
☒	☐	The statistical test(s) used AND whether they are one- or two-sided<br>*Only common tests should be described solely by name; describe more complex techniques in the Methods section.*
☒	☐	A description of all covariates tested
☒	☐	A description of any assumptions or corrections, such as tests of normality and adjustment for multiple comparisons
☐	☒	A full description of the statistical parameters including central tendency (e.g. means) or other basic estimates (e.g. regression coefficient) AND variation (e.g. standard deviation) or associated estimates of uncertainty (e.g. confidence intervals)
☒	☐	For null hypothesis testing, the test statistic (e.g. F, t, r) with confidence intervals, effect sizes, degrees of freedom and P value noted<br>*Give P values as exact values whenever suitable.*
☒	☐	For Bayesian analysis, information on the choice of priors and Markov chain Monte Carlo settings
☒	☐	For hierarchical and complex designs, identification of the appropriate level for tests and full reporting of outcomes
☒	☐	Estimates of effect sizes (e.g. Cohen's d, Pearson's r), indicating how they were calculated

Our web collection on statistics for biologists contains articles on many of the points above.

Software and code

Policy information about availability of computer code

Data collection	Topspin 4.0.6 (Bruker Biospin), MXCuBE Qt4 v 2.3
Data analysis	SEDFIT 15.1, Hydropro 10, NMRPipe 10.9 Rev 2021.258.11.26 64-bit, CcpNmr 2.5/3, ChemEx 2022.1.0, RStudio 2021.09.0 Build 351, Microsoft Excel Version 16.78.3, ChimeraX 1.6.1, Alphafold 2.3, ARIA 2.3.3, TALOS-N 4.12, Coot 0.9.8.91, Phenix 1.20.1_4487, XDSME 0.6.6.0, SMILE Version 2.1 Revision 2019.337.11.19, REFMAC 5.8.0091

For manuscripts utilizing custom algorithms or software that are central to the research but not yet described in published literature, software must be made available to editors and reviewers. We strongly encourage code deposition in a community repository (e.g. GitHub). See the Nature Portfolio guidelines for submitting code & software for further information.

Data

Policy information about availability of data

All manuscripts must include a data availability statement. This statement should provide the following information, where applicable:

- Accession codes, unique identifiers, or web links for publicly available datasets
- A description of any restrictions on data availability
- For clinical datasets or third party data, please ensure that the statement adheres to our policy

NMR chemical shift assignments of ExbDSm, peri are deposited at the Biological Magnetic Resonance Data Bank under the accession code BMRB 34826. The NMR protein structure of ExbDSm, peri and the crystal structure of ExbDEc, ΔNIBS with TonB peptide are deposited at the Protein Data Bank under the accession codes

8PEK and 8P9R respectively. The authors declare that any other data supporting the findings of this study are available within the article and in its Supplementary Information, or from the authors upon request. Source data are provided with this paper. This approach ensures efficient data sharing, balancing the resources required for data curation with the anticipated frequency of data access requests.

Research involving human participants, their data, or biological material

Policy information about studies with human participants or human data. See also policy information about sex, gender (identity/presentation), and sexual orientation and race, ethnicity and racism.

Reporting on sex and gender	N/A
Reporting on race, ethnicity, or other socially relevant groupings	N/A
Population characteristics	N/A
Recruitment	N/A
Ethics oversight	N/A

Note that full information on the approval of the study protocol must also be provided in the manuscript.

Field-specific reporting

Please select the one below that is the best fit for your research. If you are not sure, read the appropriate sections before making your selection.

☒ Life sciences ☐ Behavioural & social sciences ☐ Ecological, evolutionary & environmental sciences

For a reference copy of the document with all sections, see nature.com/documents/nr-reporting-summary-flat.pdf

Life sciences study design

All studies must disclose on these points even when the disclosure is negative.

Sample size	No statistical method was used to determine sample sizes and no sample size calculation was performed.<br>NMR: The sample size for the NMR part of the study was determined by the diversity of experiments required to achieve the study's objectives, as detailed in Table S2. Each unique sample was used for specific purposes, including assignment, relaxation measurements, and interaction studies. The table lists the different protein samples, their concentrations, labeling, and the specific NMR experiments they were used for. The number of samples was dictated by the need for a comprehensive understanding of the protein's structure and dynamics, rather than statistical power. However, in many NMR studies, due to the long acquisition times and stability of samples, it's common practice to run each unique sample only once, particularly if the aim is to determine the structure or dynamics of a protein where variability is not expected across identical samples.<br>In vivo: For the in vivo mutation experiments, triplicates (n=3) for each bacterial strain were chosen to adhere to standard practices for biological replicates in microbiological research. This sample size allows for the detection of growth differences and statistical analysis of the results, ensuring robust and reproducible data.
Data exclusions	NMR: Residues that exhibited peak overlap in the NMR spectra, which could not be reliably assigned or quantified, were excluded from the analysis.<br>In vivo: No data were excluded; all measurements were included as per the experimental design.
Replication	NMR: NMR 2D fingerprint spectra were recorded between each long 3D experiment to ensure sample consistency over time. Due to the non-destructive and highly reproducible nature of NMR spectroscopy, each set of experiments (e.g., assignment, relaxation measurements) was performed once per sample. Replication in the traditional sense does not apply as each NMR measurement is inherently reproducible if the sample remains stable, which we confirmed via the fingerprint spectra. All NMR experiments were successful on the first attempt, with no need for repetition due to sample instability or equipment failure.<br>In vivo: Each strain was cultured in triplicate to provide biological replicates for the growth assays.
Randomization	NMR: Randomization is not applicable to the experimental design of this NMR spectroscopy study, as the experiments conducted are deterministic and based on the inherent properties of the protein samples.<br>In vivo: Cultures were randomly assigned to wells in the microplate to minimize positional effects during incubation and measurement.
Blinding	NMR: Blinding was not employed in this study as the analysis of NMR data is objective and based on spectral parameters and computational models that do not benefit from subjective interpretation.<br>In vivo: Blinding was not implemented, as the experimental endpoints were objective measurements of OD600nm.

Reporting for specific materials, systems and methods

We require information from authors about some types of materials, experimental systems and methods used in many studies. Here, indicate whether each material, system or method listed is relevant to your study. If you are not sure if a list item applies to your research, read the appropriate section before selecting a response.

Materials & experimental systems

n/a	Involved in the study
☒	☐ Antibodies
☒	☐ Eukaryotic cell lines
☒	☐ Palaeontology and archaeology
☒	☐ Animals and other organisms
☒	☐ Clinical data
☒	☐ Dual use research of concern
☒	☐ Plants

Methods

n/a	Involved in the study
☒	☐ ChIP-seq
☒	☐ Flow cytometry
☒	☐ MRI-based neuroimaging

Plants

Seed stocks	N/A
Novel plant genotypes	N/A
Authentication	N/A
